# The Chickahominy T.R.U.T.H. (Trust, Research, Understand, Teach, and Heal) Project—A Tribal Community–Academic Partnership for Understanding the Impact of Structural Factors on Perceived Cancer Risk in Rural Virginia

**DOI:** 10.3390/ijerph21030262

**Published:** 2024-02-24

**Authors:** Katherine Y. Tossas, Bianca D. Owens, Savannah Reitzel, Jacqueline Knight Wilt, Paula Tatiana Rivera Mejía, Rachel Hunley, Haley Groesbeck, Hillary Boucher, Katelyn Schifano, Susann L. Brown, Dana Adkins, Stephen Adkins, Maria D. Thomson

**Affiliations:** 1 Department of Social and Behavioral Sciences, School of Population Health, Virginia Commonwealth University, 830 East Main Street, Richmond, VA 23219, USA; owensb2@vcu.edu (B.D.O.); riveramejipt@vcu.edu (P.T.R.M.); mthomson2@vcu.edu (M.D.T.); 2 Department of Epidemiology, School of Population Health, Virginia Commonwealth University, 830 East Main Street, Richmond, VA 23219, USA; 3 Comprehensive Cancer Center, University of Puerto Rico, P.O. Box 363027, San Juan, PR 00931, USA; 4 Massey Comprehensive Cancer Center, Virginia Commonwealth University, 417 North 11th Street, Richmond, VA 23219, USAhaley.groesbeck@vcuhealth.org (H.G.); connollyh@vcu.edu (H.B.);; 5 Chickahominy Indian Tribe Inc., 8200 Lott Cary Road, Providence Forge, VA 23140, USA; susann.brown@chickahominytribe.org (S.L.B.); dana.adkins@chickahominytribe.org (D.A.);

**Keywords:** cancer risk disparities, landfill leachates, Native American cancer risk, community–academic partnership, structural violence, Policies, Systems, and Environments (PSE), well-water contamination, cancer education outreach, community health disparities, environmental justice

## Abstract

In 2022, the Virginia Chickahominy Indian Tribe partnered with Virginia Commonwealth University Massey Comprehensive Cancer Center to investigate concerns about a potential cancer cluster near a local landfill. While investigating cancer clusters is complex due to long latency and multifactorial causes, the community’s concerns about structural factors driving cancer risk warrant exploration. Thus, the Chickahominy T.R.U.T.H. (Trust, Research, Understand, Teach, and Heal) Project was created as a community–academic partnership to (1) identify structural factors and barriers associated with perceived cancer risk and care; (2) assess cancer knowledge, care access gaps, and perceived risks, including testing private and community water sources; (3) develop and deploy culturally tailored cancer education and resource navigation, including groundwater safety education, policies, and remediation. We will conduct 150 in-person interviews and water tests among residents within a four-mile radius of the landfill, and deploy 1000 structured questionnaires among Charles City County residents. In this paper, we provide an overview of the ongoing project design, development, and progress in support of the project’s objectives. This collaborative investigation aims to address cancer health disparities, enhance research and health policy advocacy, and honor the sacred knowledge of an underserved community, laying the groundwork for a long-term partnership to guide future research questions.

## 1. Introduction

**Figure 1:**
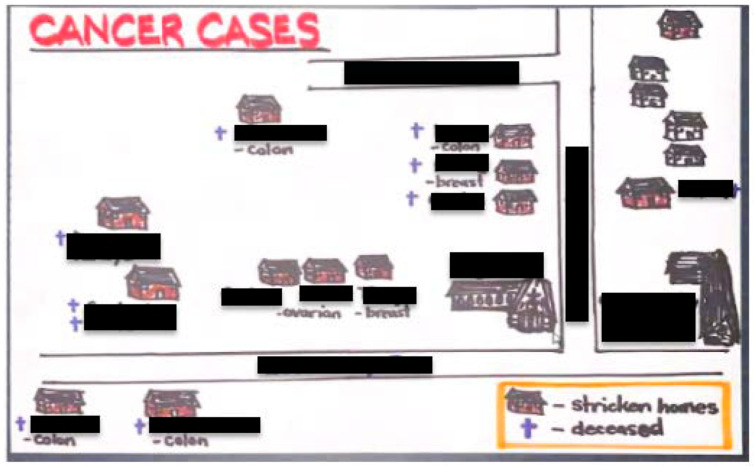
Hand-drawn map of ~15 cancers reported among Chickahominy Tribal members, within a one-mile radius of a local landfill (not shown), from the year 2000 to date.

In January of 2022, Virginia Chickahominy Indian Tribe (CIT) leaders approached Virginia Commonwealth University (VCU) Massey Comprehensive Cancer Center (MCCC) researchers about a potential community cancer cluster (Video S1: VCU State of the University: Chickahominy T.R.U.T.H. Project: https://www.youtube.com/watch?v=KgHWRBwNvdo (accessed on 8 September 2023)). Armed with a hand-drawn map (Figure 1), they showed over 15 cancers diagnosed within a one-mile radius of a local landfill. The privately owned landfill opened in 1990 [1] and cancers began surfacing in the 2000s. Most cancer types reported (breast, ovarian, prostate, colorectal, kidney) are those linked in the scientific literature to water pollution such as landfill leachate—the liquid that percolates through degrading waste. Leachate is a known source of toxic heavy metals and endocrine disruptors like bisphenol-A and is associated with elevated cancer incidence among residents near contaminated sites [2,3,4]. This information alone satisfies three critical epidemiological causal criteria: plausibility (there is a reasonable pathway linking the outcome (cancer) with the exposure (leachates)), temporality (the exposure to leachates preceded the cancers), and consistency of evidence (consistent scientific evidence supports a link between landfill leachates and cancers) [5]. The Virginia Cancer Registry confirmed that 35% (143 of the 406) of the cancer cases diagnosed in Charles City County from 2013 to 2019 occurred within a four-mile radius of the landfill. Further, standardized incidence ratios (SIRs) showed significantly elevated cancer incidence rates in specific age groups. The most pronounced excesses were observed in the age groups 50–54 (SIR = 1.73; 95% CI: 1.13–2.53), 60–64 (SIR = 1.64; 95% CI: 1.23–2.15), and 65–69 (SIR = 1.43; 95% CI: 1.08–1.84). These SIR values, notably exceeding 1.0, indicate a higher incidence of cancer cases than would be expected based on regional or national averages. However, there are challenges involved in investigating cancer clusters due to cancer’s long latency and complex, multifactorial etiology. We can, however, investigate the contribution of structural factors (i.e., the built environment, local policies, and healthcare access/resources) on cancer diagnoses and subsequent care outcomes.

The CIT, one of seven federally recognized native tribes in Virginia (VA), is no stranger to the historic, systemic, and racist structures that impact health [6,7]. Their approximately 836 Tribal members congregate predominantly within a five-mile radius of their Tribal Center in Charles City County—a majority (racial/ethnic) minority (44% non-Hispanic Black, 43% non-Hispanic White, 6% American Indian and Alaska Native) county whose residents live on average five years less than other Virginians (75 versus 80 years of age), and which ranks near the bottom in VA for health outcomes (113th of the 133 VA cities and counties) [8]. This trend of worse health outcomes is also seen in cancer nationally, where American Indians and Alaska Natives face a higher burden of infection-related cancers. The delayed reductions in cancer incidence trends can be attributed, in part, to delayed smoking cessation efforts and a slow uptake of colorectal cancer screening in this community. Consequently, this has led to unchanged or even increased cancer incidence and mortality rates for these populations [9]. These observed outcomes are probably connected to systemic obstacles in healthcare access, as evidenced by the county having merely two physicians for approximately 7000 residents. Additionally, it has the fourth highest rate of uninsured adults in Virginia, at 17%, compared to the state average of 12% [10].

Thus, we proposed a community–academic partnership between the CIT and MCCC, jointly named The Chickahominy T.R.U.T.H. (Trust, Research, Understand, Teach, and Heal) Project, reflective of our commitment to build a trustworthy, equitable partnership, to research structural and individual-level contributors to cancer risks, to understand such risks based on evidence-based approaches and the community’s intelligence, and to share the findings using culturally tailored cancer education through high-tech and high-touch approaches that integrate the traditional knowledge system embedded in the Chickahominy tradition—all of which aim toward collective healing via truth seeking and truth telling. Specifically, we proposed using the Policies, Systems, and Environments (PSE) [11] seven-step change strategy as a framework to accomplish the following aims: (1) identify structural factors and barriers associated with perceived cancer risk and cancer care; (2) assess cancer knowledge and access to care gaps as well as perceived risks, including testing individual (wells) and community (creeks) water sources; (3) develop and deploy culturally tailored cancer education and resource navigation, including groundwater safety education, policies, and remediation.

## 2. Materials and Methods

Theoretical Framework: The overarching theoretical framework informing the study design is the PSE framework [11]. The PSE focuses on the identification and implementation of community- and population-level initiatives that offer long-term change by leveraging or embedding interventions within existing systems. The PSE allows users to identify how and where existing structures at the policy, system, and environmental levels require change, thus shifting focus away from individual drivers to community- or society-level drivers. Furthermore, the PSE was used to develop and align the interview guide and survey questions to interrogate factors influencing perceptions of cancer risk and access to care in the community.

Study Design: Our study employs a concurrent mixed-methods design, incorporating individual interviews, the testing of private well water and surface water, and community surveys to address study objectives one and two. Furthermore, we adopt a community-engaged approach [12,13], with the Chickahominy Tribal leadership members acting as equal partners in all stages of the study’s design, execution, analysis, evaluation, and resource allocation. Additionally, we provide training to community members (excluding Tribal co-investigators) involved in recruitment and data collection, playing a crucial role in creating lasting communication materials in line with the objectives of aim three.

Establishment and Training of the T.R.U.T.H. Health Brigade: Following principles of community-engaged research [12,13], the study leaders (both community and academic) agreed that it was critical to engage community members as part of the research team. To this end, we created the T.R.U.T.H. Health Brigade—a coalition of community-based advocates, including high-school students, as well as VCU-affiliated graduate students and staff. This group operated in pairs responsible for identifying, recruiting, and interviewing participants, and disseminating the survey within the community. To build rapport, all Brigade members (also referred to as Health Brigadiers) were trained together. Training consisted of 6 h of combined in-person, online (via Zoom), and asynchronous sessions. The in-person sessions were used to introduce the community-based advocates and VCU-affiliated personnel, to teach them about study-specific procedures, and so that they could practice their interviewing skills. The Zoom and asynchronous sessions were used to cover logistic procedures for data collection and storage, as well as to complete training in research ethics. All T.R.U.T.H. Health Brigade members were required to complete the Collaborative Institutional Training Initiative, Good Clinical Practice Training for Social and Behavioral Research through the Society of Behavioral Medicine, or the University of Illinois Chicago CIRTification for human subjects research protection training prior to beginning study recruitment. Specific topics covered during these sessions encompassed: project purpose and background, various research methodologies, well-water testing, professionalism, fundamental principles of community–academic partnerships, and study conduct in adherence to protocol (i.e., recruitment, consent procedures, qualitative interviewing techniques, data safety, personal safety). The community-based Health Brigadiers were identified using community notices, word of mouth, social media, and brief presentations about the study delivered by both academic and Tribal investigators. Community-based Health Brigadiers are compensated for their time in the amount of up to USD 250, dependent on the length of their engagement. For example, a community-based Health Brigadier who completes all training requirements, conducts six interviews, and participates in four recruitment activities receives the full USD 250. VCU-affiliated personnel do not receive remuneration from this project.

Sample Identification and Recruitment: Two separate participant samples are being recruited; first, we are conducting *n* = 150 semi-structured interviews and well-water tests with adults (18+ years of age) who live within 4 miles (6.44 km) of the local landfill. This radius was selected as the focal area for water sampling based on multiple factors, including geographical data and environmental considerations, as well as suggestions from the Tribal Environmental Director. Upon further community query, the Chickahominy leaders amended their prior list from 15 to 60 households among their residents diagnosed with cancer. These residences are located within a 4-mile radius. Further, a review of the literature revealed variability in the recommended isolation distances between landfills and groundwater sources, contingent on aquifer composition. These distances range from 106 m to 5.46 km for sand aquifers, 292 m to 13.5 km for gravel aquifers, and 2.4 to 58.7 km for coarse gravel aquifers, considering potential leachate migration to groundwater [14]. Aquifers in Charles City County exhibit a diverse mix of gravel, sand, silt, and clay, necessitating an inclusive approach in defining the study area [15]. Additionally, the landfill’s proximity to key geographical landmarks, such as the Chickahominy Tribal Center (approximately 2.47 km) and the closest groundwater source, Bradley Creek Run (809.4 m), which connects to the Chickahominy River, supports the appropriateness of this radius.

Second, we are currently administering a survey to *n* = 1000 adult community members who live anywhere in Charles City County. The determination of the survey sample size was influenced by collaborative discussions with key stakeholders, notably the Chickahominy Health District. This partnership significantly shaped our approach, with the Health District strongly supporting the collection of 1000 surveys. This number is not arbitrary; it represents a substantial 18% of the adult population within the county (as of 7/2022 census estimates) [8]. The rationale behind targeting such a considerable sample size stems from the Health District’s recognition of the exceptional value of comprehensive data. They perceive this study as an unprecedented opportunity to obtain a rich and extensive dataset, one that they, given their limited budget and personnel resources, would not be able to collect independently. This collaborative effort aligns with the objectives of both the tribe and the Health District, who are united in their goal to acquire detailed insights into the health status of their community. Furthermore, the collection of such extensive data, which is a cornerstone of this project, will directly inform and enhance their needs assessment processes. These data will be shared with the Chickahominy Health District, underscoring our mutual commitment to this endeavor and the value placed on our collaborative relationship.

The interviewing and well-water testing is restricted to those living in a smaller geographical area due to costs associated with purchase and analysis of the water test kits (approximately USD 400/kit). We are focusing these assessments, which will represent approximately 10% of households, in the area of the county initially identified by our Tribal partners as being an area of concern. Households in which any member has received any cancer diagnosis in the past twenty years were initially prioritized, but this was not an inclusion criterion. Participants are identified using several different methods. Study-specific recruitment materials include a study website, posters, flyers, and social media posts. We have advertised in local newspapers and businesses, mailed postcards to all households in the county, and participate in monthly community events. T.R.U.T.H. Health Brigade members assist with word-of-mouth recruitment and providing study contact information to individuals who are interested in participating. Finally, we use snowball sampling techniques with those who participate in either the interviews or the survey. Individuals who are interested in participating can contact the study team directly via web request, telephone, or email.

Interviews: A semi-structured interview guide with standardized probes was developed with input from academic and Tribal investigators. After its development, the guide was subjected to pretesting by the T.R.U.T.H. Health Brigade members, comprising community-based advocates, who provided feedback on question clarity and proposed the inclusion of several additional probes. The guide was designed to support a 60–90 min interview and asked participants to describe their attitudes, beliefs, and experiences in relation to cancer in both their family and in the wider community. Interviews are conducted in person at the participant’s home at a convenient day/time. One member from each household is identified to complete the interview and be the primary voice, although other family members are often present during the interview. Interviews are audio-recorded and transcribed verbatim. Each interview is conducted by two Health Brigadiers, ideally consisting of one community-based advocate paired with one VCU-affiliated personnel. After completion of an interview, the two Health Brigadiers members complete a fidelity checklist and a water sample is collected from the home. The well-water sampling kit, valued at USD ~400, is provided to the household free of charge.

Private Well-Water Testing. Private well-water samples are obtained from each house after completing the semi-structured interview. Water testing is completed using a direct-to-consumer product (Safe Home Ultimate Drinking Water Test Kit) [16], which tests for over 200 potential contaminants classified by the U.S. Environmental Protection Agency as likely to be carcinogenic in humans. Results are mailed from the company to the participants. The study team does not engage in interpretation of the water testing results. The Chickahominy Tribal Center and Health District offices have lists of resources available to persons who require additional information or assistance managing issues associated with private wells. The study team is provided with the raw results of the water tests for integration into data analysis. The site of each well test is further mapped using ArcGIS version 2.8.

Community Survey: A survey of *n* = 1000 is currently being administered to the wider Charles City County community to collect information about *medical mistrust*, *perceived cancer risk*, *perceived occupational and environmental exposures*, and *healthcare access*. Each construct is measured using robust, commonly employed, and psychometrically acceptable measures. *Medical mistrust* is measured using 7 items on a 5-response-item Likert scale ranging from 1 (strongly disagree) to 5 (strongly agree) (e.g., “Health care organizations have sometimes done harmful experiments on patients without their knowledge”, “Sometimes I wonder if health care organizations really know what they are doing”). Responses are coded so that higher scores indicate higher mistrust [17]. *Perceived cancer risk* is assessed by using selected existing items from two validated measures. First, 6 items from the Health Information National Trends Survey (HINTS) are used to measure general cancer risk perceptions (e.g., “It seems like everything causes cancer”, “I worry a lot about cancer”) [18]. Additionally, we made slight adjustments to the wording of 2 items originally from the Perceptions Breast Cancer Risk questionnaire to make them applicable to an individual’s perceived risk of all types of cancer (e.g., “How likely do you think it is that you will develop cancer in the future?”) [19]. *Perceived occupational and environmental exposures* questions were adapted from a scale developed for industry workers to appraise their exposure to 5 common carcinogens including secondhand tobacco smoke, radon, substances (e.g., asbestos, arsenic, lead, coal soot/tar, nickel, and chromium), air pollution, and water pollution. Participants are asked if they believe they have been exposed to each of these carcinogens, specifying if the exposure occurred at their home, work, and/or within the community [20]. These constructs were chosen to assist with the identification of structural factors and barriers associated with perceived cancer risk and cancer care. *Healthcare access* is measured using 10 items from the National Health Interview Survey, modeling a prior study of American Indian healthcare utilization [21]. These items ask participants about their access to a regular location for preventive and sick care, their worries about healthcare costs, and delays they have experienced in seeking healthcare (e.g., not being able to get through on the phone, not being able to get an appointment soon enough, waiting too long at the doctor’s office, the office not being open when they could go, and having no transportation). Finally, we administered questions to gather information on additional cancer risk factors, including but not limited to family history of cancer, HPV vaccination status, obesity, harmful tobacco use, excessive alcohol use, and exposure to carcinogenic substances. Survey participants have the option to complete the survey online or in-person and receive a gift card worth USD 10 (electronic or physical card).

Proposed Data Analysis: We will implement a comprehensive mixed-methods approach to gain insights from the data. We will conduct descriptive and associative analyses to examine the prevalence and distribution of a range of water contaminants. The 200 analytes detected with our testing kit will be classified into three distinct categories: aesthetic (compounds influencing the water’s taste, smell, or appearance), appliance-related (substances potentially damaging to appliances and plumbing fixtures), and health-related (elements with potential adverse health effects). We will use spatial analysis to assess any discernible patterns in the distribution of these analytes. We will also measure correlations between the categories of contaminants and various participant-related factors. These factors include demographic data and self-reported characteristics, encompassing perceptions of health, healthcare access, healthcare mistrust, and cancer fears. We will also explore associations between demographic and participant characteristics with healthcare mistrust scales and cancer concerns. This will provide insights into how socio-demographic factors might influence health perceptions and fears related to cancer.

Qualitative data will be analyzed iteratively using a grounded theory approach [22]. This method involves several trained research staff breaking down the data into discrete units and coding them into categories that reflect the language and concepts of the study participants. As per Corbin and Strauss [22], we will reconstruct the categories used by participants to conceptualize their experiences and worldview and develop theoretical insights into their beliefs, attitudes, and experiences in relation to cancer and access to care. By continually comparing specific units in the data, we will refine these concepts and use them to generate new and unexpected themes and variables. A code book was developed using a first round of 10 transcripts and includes code definitions and examples. The fit of the original codes will be examined along with the need to modify or add new codes to assess for data saturation (the point where no new themes are identified). Inter-rater reliability will be examined regularly using interclass correlations to ensure that all interviews are coded consistently and reliably. For items on which the coders have poor agreement (i.e., ICC < 0.9), the coding rules will be reviewed and revised with additional training provided. The coding team will meet weekly and 10% will be randomly selected for double coding to assure against rater drift. Once completed, we will engage community members from the Chickahominy Tribe and the broader Charles City community for ground truthing and providing additional insights into our data findings.

Data analyses of the qualitative and quantitative data will occur simultaneously, with both separate and combined analyses planned. The integration of quantitative data from our descriptive and associative analyses with qualitative insights from the grounded theory approach will enable a robust, mixed-methods analysis. This comprehensive strategy, alongside the gathered community intelligence, will not only provide nuanced information on the spatial distribution and potential impacts of water contaminants but also potentially offer some contextualized understanding of how these contaminants intersect with health perceptions and behaviors.

Developing and Deploying Culturally Tailored Cancer Education: Working in partnership with Tribal leadership, the T.R.U.T.H. Health Brigade members, and resources from VCU MCCC, we will produce a series of communications (e.g., short videos, maps, and infographics) that merge thematic data, numeric data, and water contaminant reports to inform and educate community members. The primary findings of this study will form the basis of these communications, informing which topics, content, communication channels, and community-specific tailoring processes are prioritized. Additionally, we will convene community members, including Tribal members and Health District representatives, to discuss the findings, share reactions, concerns, experiences, and provide suggestions for research needs and priorities. An evaluation will be conducted to assess the knowledge change after the dissemination of the communication materials.

## 3. Results

Community Engagement and Health Brigadiers: The core objectives and methods laid out at the inception of our project have formed a sturdy foundation, enabling us to address a spectrum of cancer-related issues, foster community engagement, amass comprehensive data, and deliver culturally tailored education and support. Through the training of Health Brigadiers, the T.R.U.T.H. Project also aligns with the CIT’s overarching goal to develop healthcare career pipeline training programs, thereby bolstering the representation of non-dominant cultural narratives in the healthcare sector.

To date, we have successfully recruited and trained an enthusiastic cohort of 11 community-based T.R.U.T.H. Health Brigade members, all of whom are dedicated advocates from the Chickahominy community. By employing a joint approach to introduce the project, we have achieved positive community awareness, support, and successful participant identification and recruitment. The biggest challenge has been maintaining regular participation of the Brigade members. For example, transportation to interviews can be a challenge for graduate students as many participants live in rural areas of the county; for community members, balancing school and work responsibilities with data collection has been a challenge. However, as we navigate the challenges of maintaining consistent engagement across our Brigade members, we are mindful of preventing overworking and burnout among a select few. To address this concern, we have implemented ongoing quarterly training sessions for new Health Brigade members. Furthermore, we have refined our options and associated honorariums for community Brigade members, thereby offering them greater flexibility and alignment with their interests and availability.

Data Collection Progress: Our data collection efforts have made significant headway. To date, the Health Brigadiers have actively contributed toward the completion of 60 in-person interviews and at-home water tests. It is noteworthy that our sampling strategy, initially based on snowball sampling, has yielded a majority Native American sample (66%) thus far, which we expect might evolve in the future to a more representative distribution. Additionally, we have effectively collected 448 community surveys. These survey results indeed indicate an evolving demographic shift, now more closely mirroring the racial and ethnic distribution of Charles City County, with 23% White, 39% Black, and 34% Native American respondents (compared to the county’s racial/ethnic distribution of 46% White residents, 43% Black residents, and 7% American Indian and Alaska Native residents). Descriptive and associative results will be released and reported in a future manuscript at the conclusion of the project.

Qualitative Data Analysis and Stakeholder Engagement: The pivotal next phase of our project involves the commencement of qualitative data analysis. Our graduate research assistants, who also serve as Health Brigade members, will lead this analysis. Once half of the qualitative interviews have been collected and analyzed, preliminary findings will be shared with T.R.U.T.H. Health Brigade community advocate members and Tribal investigators. These crucial conversations will offer an opportunity to evaluate our progress, identify the next steps, and determine whether any adjustments or expansions are required in our data gathering plan.

Water Testing and Health Impact: One of the key deliverables of our project is the provision of EPA-certified water testing for local creeks and families affected by cancer in the community. We have successfully completed well-water testing and promptly conveyed the results to 60 households. Importantly, no results have indicated an immediate need for attention or remediation. These results not only serve as a valuable dataset for hypothesis-driven research but also significantly contribute to the ongoing efforts of the Chickahominy City Health District in mapping private wells and conducting comprehensive assessments of health needs.

## 4. Discussion

Addressing Community Health Disparities: The T.R.U.T.H. Project has embarked on a multifaceted journey to address pressing cancer-related concerns within the CIT community. As we reflect on our progress and the implications of our work, it is imperative to contextualize our efforts within the broader landscape of systemic health disparities. The stark reality is that Charles City County grapples with a multitude of systemic health disparities, a fact underscored by our findings. Its sociodemographic and healthcare landscape coalesce to create an environment where healthcare is not only limited in supply but also fraught with financial barriers, thus intensifying the disparities experienced by the CIT and the broader community.

The Role of the T.R.U.T.H. Project: In the face of these formidable challenges, the T.R.U.T.H. Project has emerged as a beacon of hope and change. Our collaborative efforts, featuring dedicated Brigade members, committed researchers, and engaged community stakeholders, are emblematic of the positive momentum generated by this initiative. Indeed, Brockie et al. recently outlined critical strategies for developing culturally safe research partnerships with Native American communities, including increasing research capacity and shared project governance [23], features which have been foundational to the T.R.U.T.H. Project.

The comprehensive data we are amassing through our project holds the potential to serve as a catalyst for transformative change. By shedding light on the intricacies of cancer perceptions, access to care, and environmental factors, our work contributes not only to understanding cancer risks but also to unveiling the broader structural factors that underpin health disparities. Furthermore, this knowledge is pivotal in informing evidence-based interventions and policies that can begin to rectify the systemic issues at play.

Through the dedication of our Brigade members, the unwavering commitment of our researchers, and the active participation of community stakeholders, we have made significant strides in our project. Training and mobilizing Health Brigadiers from within the Chickahominy community not only enhances our research capacity but also aligns with the CIT’s goal of building a healthcare career pipeline, amplifying the representation of non-dominant cultural narratives.

Despite the challenges of maintaining consistent engagement among our Brigade members, we have adapted and refined our strategies to ensure that community members can participate meaningfully, on their terms, and with respect to their interests and availability. We have successfully conducted interviews and at-home water tests, collected an extensive set of community surveys, and initiated vital water testing for local creeks and affected families.

We also believe one of the most profound and distinctive aspects of the Chickahominy T.R.U.T.H. Project lies in its commitment to amplifying the voices of the CIT and, by extension, Native American communities at large. The stark reality is that indigenous populations, including the CIT, have historically been marginalized and rendered invisible in the realm of scientific research and public health initiatives. This invisibility has perpetuated a cycle of systemic neglect, wherein the unique challenges, experiences, and health disparities faced by Native American communities often go unaddressed. To effectively address these challenges, there must also be acknowledgement of the substantive cultural and linguistic diversity between Native American communities; as noted in the 2022 American Cancer Society report, there are more than 574 federally recognized tribes in the US [24,25], with the CIT being one of them. The T.R.U.T.H. Project combines a shared leadership approach that enables the identification of structural barriers as told by our partners, situated within their unique historical experience and cultural priorities [26].

The CIT community, like many Native American communities, has endured the erasure of its narratives and the omission of its perspectives from academic research, healthcare policy making, and public discourse. This exclusion has profound implications for the health and well-being of these communities, as their specific needs and concerns remain largely unexamined and unattended.

The T.R.U.T.H. Project recognizes that the invisibility of the CIT within the broader research landscape perpetuates health disparities and exacerbates the challenges they face. By actively engaging with the CIT community, our project serves as a powerful counterforce to this historical invisibility. We acknowledge that the health disparities faced by the CIT extend beyond cancer-related concerns, touching upon broader issues of health equity, social justice, and community empowerment.

Our commitment to centering the voices of the CIT goes beyond this research; it is a moral and ethical imperative. The knowledge and insights we gather through this project not only contribute to understanding cancer risks but also serve to unveil the systemic factors that underpin health disparities within the community. By bringing these issues to light and fostering community-driven solutions, we aim to dismantle the cycle of neglect and invisibility that has persisted for far too long.

Promoting Health Equity and Well-Being: The T.R.U.T.H. Project is not merely an isolated research endeavor; it is a driving force for promoting health equity and improved well-being within the Chickahominy community and Charles City County at large. Our project is rooted in a commitment to building a trustworthy, equitable partnership with the CIT, one that respects the community’s intelligence and values while aiming for collective healing through truth seeking and truth telling.

As we move forward, it is our hope that the insights and data gleaned from this project will empower the community and its stakeholders to advocate for change, to demand equitable access to healthcare, and to address the underlying structural factors that perpetuate health disparities. In doing so, we aspire to contribute to a brighter and healthier future for the Chickahominy community, where health equity is no longer an aspiration, but a tangible reality.

## 5. Conclusions

The Chickahominy T.R.U.T.H. Project represents a transformative journey toward addressing the multifaceted challenges faced by the Chickahominy Indian Tribe (CIT) community in Charles City County. Our collaborative and community-centered approach, as outlined in the preceding sections, lays the foundation for meaningful change and progress in various dimensions of health, well-being, and community resilience.

Our goal is to ensure that the narratives, concerns, and experiences of the CIT community are not only acknowledged but celebrated as vital contributors to the broader discourse on health equity and social justice. As we continue this journey, our hope is that our efforts will inspire broader discussions, advocate for equitable access to healthcare, and ultimately serve as a catalyst for transformative change, not only within the CIT community, but for all marginalized and under-represented communities. We aspire to create a future where health equity is not a distant aspiration but a lived reality for all, ensuring that no voices are left unheard or invisible. 

## Data Availability

A de-identified dataset with limited access may be made available upon reasonable request to the primary investigator, subject to approval from the community advisory board. The availability of the data is contingent upon ensuring the privacy and confidentiality of the study participants and aligning with the principles of community-driven research.

## Supplementary Materials

The following supporting information can be downloaded at: https://www.youtube.com/watch?v=KgHWRBwNvdo (accessed on 8 September 2023), Video S1: VCU State of the University: Chickahominy T.R.U.T.H. Project.
